# Torsion and Axial Deformations of Chalcogen Helical Chains (S, Se, Te): First Principles Calculations Using Line Symmetry Groups

**DOI:** 10.3390/nano15070505

**Published:** 2025-03-27

**Authors:** Robert A. Evarestov, Vitaly V. Porsev, Dmitry D. Kuruch, Polina Yu. Cherezova

**Affiliations:** Quantum Chemistry Department, St Petersburg State University, St. Petersburg 199034, Russia; v.porsev@spbu.ru (V.V.P.); d.kuruch@spbu.ru (D.D.K.); pol.cherezz@mail.ru (P.Y.C.)

**Keywords:** sulfur, selenium, tellurium, line symmetry groups, helical chains, DFT

## Abstract

The atomic structure, electronic, phonon, and optical properties of chalcogen helical chains (S, Se, Te) were studied using line symmetry groups and DFT calculations. The whole possible range of torsion deformations (from 0° to 180°), as well as the range of axial deformations (from 0.6 to 1.6) were considered. For the studied chains, the atomic and electronic structures at the energy minima were found. It was shown that for the considered chalcogen chains, the minimum of electronic energy is in the region of rotation angles ~103–107°. The electronic structure of all chains was considered in the helical Brillouin zone, which made it possible to trace its evolution up to the extreme torsional deformations: 0° (linear chain) and 180° (zigzag chain). A method for obtaining the dispersion of phonon states in the helical Brillouin zone has been developed based on the results of calculations by the CRYSTAL17 program. This allowed us to trace the evolution of phonon dispersion curves under torsion deformations up to their extreme values. Based on the known selection rules for helical polymers, the energies of optical, IR, and Raman transitions were obtained. This allows one to predict the optical properties of atomic chalcogen chains—both in a free state and inside carbon nanotubes.

## 1. Introduction

Among all chalcogens, tellurium, selenium, and some phases of sulfur bulk crystals can be represented as ensembles of helical atomic chains [1,2,3]. The chains are linked together by van der Waals forces. For tellurium and selenium, crystals of such a structure are the most stable phases. For sulfur, there is a high-pressure helical sulfur phase, S-II [3]. The mentioned crystals are described by the chiral (non-symmorphic) space symmetry group *P*3_1_21 [1,2]. The order of the helical axis of the atomic chains is equal to 3. Besides stable phases, the high-pressure phases with symmetry *I*4_1_/*acd* have also been obtained for selenium, Se-II’ [4], and sulfur, S-III [5]. These phases can be represented as ensembles of helical atomic chains, but with order 4 of the helical (screw) axis.

It can be expected that for selenium and tellurium (possibly also sulfur), the most natural nanostructure is nanorod, i.e., mono-periodic nanocrystal consisting of a finite number of formally infinite helical atomic chains. Indeed, the structure of experimentally synthesized selenium [6] and tellurium [7] nanorods consists of helical chains. Moreover, it was possible to synthesize ultra-thin tellurium nanorods, in which a deviation of the helical axis order of the nanorod from the crystallographic one was experimentally observed [8]. This deviation is due to the fact that the order of the helical axis is no longer limited by the requirement of translational periodicity in the directions perpendicular to the main nanorod axis, so it can differ from the crystallographic one [9]. The smaller the thickness of the nanorod, the greater this deviation [9].

A single helical chain of chalcogens (S, Se, Te) is not only an extremely thin nanorod, but also a helical inorganic polymer. This means that such chains are completely free of any restrictions on the order of the helical axis. This behavior makes individual chalcogen atomic chains a unique nanoobject, the physical properties of which can be changed in a wide range by simple mechanical stresses—torsion (variations in the helical axis order), axial (variations in a partial translation), or their combinations. Note that single atomic chains have been synthesized for all three chalcogens using carbon nanotubes as a container [10,11,12,13], or by incorporation into various single-crystalline zeolites [14,15,16]. According to experimental data, the atomic chain inside a carbon nanotube or zeolite interacts with the nanotube walls only by van der Waals forces and retains their structure. In addition, depending on the radius of the inner volume of the nanotube or zeolite pore, the atomic chains of chalcogens can adopt various conformations. In particular, in addition to the helical conformation, zigzag and linear conformations were experimentally found [10].

To date, the properties of single helical chains have been actively studied using theoretical methods. However, the vast majority of studies of sulfur, selenium, and tellurium chains are limited to the crystallographic order of the helical axis, i.e., *p*3_1_21 rod group [17,18,19,20,21,22,23,24,25,26,27]. In other words, the atomic chains are considered as extremely thin nanorods, without taking into account the additional degree of freedom (i.e., variations in the helical axis order). Only in a few works, in which the atomic helical chains of sulfur [28,29,30] and selenium [30,31,32] are considered as inorganic polymers, the order of the helical axis was optimized. It was shown that for both sulfur and selenium, the potential energy minimum corresponds to a value of the helical axis order equal to approximately 3.4 [30], i.e., in the middle between the crystallographic values 3 or 4. This result clearly shows that assigning a crystallographic value to the order of the helical axis of the atomic chain is a significant simplification of the real picture.

Real nano-objects may contain various defects that can significantly influence their properties. Our results were obtained for perfect infinite chains and can be considered as an initial step for further construction of more complex and real nanoobjects. In particular, the supercell method can be used to model defects, which is easier to implement for monoperiodic systems compared to bulk crystals or layers.

Our paper presents the results of high-level quantum chemical modeling of atomic chains of sulfur, selenium, and tellurium based on the theory of line symmetry groups [33]. In addition to the structural, energetic, electronic, phonon, and optical properties of the atomic helical chains in the region of energy minimum, the evolution of these properties in a wide range of torsion and axial deformations was considered.

## 2. Computational Details

The CRYSTAL17 computer code was used for our calculations [34,35]. This program is designed for high-quality ab initio calculations of atomic and electronic structure and other properties, with maximum taking into account of symmetry. The atomic basis sets used in CRYSTAL code are optimal for calculating nanosystems with reduced periodicity, in particular, nanosystems with helical symmetry [36]. At the same time, artificial three-dimensional periodicity is required in plane wave calculations of monoperiodic systems.

Present calculations were carried out using the DFT method. Among the density functionals available in the CRYSTAL17, those that best reproduce the experimental data of the most stable bulk crystal phases (α-S_8_, Se-I, and Te-I, see Table 1) were chosen for subsequent calculations of chains. Another necessary requirement was to reproduce the correct order (at zero pressure) of formation energies of known high-pressure phases of sulfur and selenium. The formation energy Δ*E*_form_ of some object defined as the energy per atom, referred to the most stable phase:

(1) $$∆E_{form}=\frac{E_{object}}{N_{object}}-\frac{E_{bulk}}{N_{bulk}}$$

Here, *E*_bulk_ and *N*_bulk_ are energy and number of atoms per periodic cell of most stable bulk crystal phase, respectively. Most stable phases of chalcogen 3D crystals are α-S_8_, Se-I, and Te-I with 32, 3, and 3 atoms per periodic cell, respectively. *E*_object_ and *N*_object_ are energy and number of atoms per periodic cell of object under consideration, respectively.

For selenium and tellurium, theoretical estimates of the exfoliation energy, *E*_exf_, of atomic chains, *E*_exf_, obtained earlier using the DFT with nonempirical van der Waals functional [26] and the semi-empirical Grimme correction [27], were also taken into account. The *E*_exf_ is equal to the formation energy of a single optimized chain with symmetry *L*3_1_21:

(2) $$E_{exf}(Se\;or\;Te)=∆E_{form}(Se\;or\;Te,\;L3_{1}21).$$

In the case of sulfur, the exfoliation energy is not well defined because the most stable phase of sulfur is an ensemble of S_8_ rings rather than of quasi-one-dimensional chains.

Overall, the best match was achieved for short-range corrected HSEsol [37] and HSE06 [38] density functionals. The HSEsol with dispersion correction D3 (version 3) [39] was used for sulfur. Unfortunately, the structure optimization of the bulk Se-I crystal resulted in an unacceptable error of 27% for the lattice parameter *a*. Therefore, selenium was calculated using HSE06 with dispersion correction D3 (version 3), resulting in only 2% error for the lattice parameter *a*. The HSEsol without dispersion correction was used in the calculation of tellurium, since calculation with its inclusion leads to a significant overestimation of the exfoliation energy, *E*_exf_ (HSEsol-D3) = 37.5 kJ/mol (see Section 3.1). The Gaussian-type atomic basis sets from [40], “Se_pob_TZVP_rev2” [41] and “Te_m-pVDZ-PP_Heyd_2005” [42] were used for sulfur, selenium, and tellurium, respectively. The results of calculations of bulk phases using the density functionals and basis sets chosen for further chain calculations, as well as known experimental data, are given in Table 1.

**Table 1:** Calculated structural, energetic, and electronic properties of some bulk phases of S, Se, and Te.

Phase	Lattice Parameters, Å	Δ*E*_form_, kJ/mol	*E*_gap_, eV
α-S_8_, *F*ddd	*a* = 10.057 (10.467) ^1^ *b* = 12.337 (12.870) ^1^ *c* = 23.945 (24.493) ^1^	0	2.77 (2.61) ^2^
S-II, *P*3_1_21	*a* = 7.258 (7.090) ^3^ *c* = 4.326 (4.302) ^3^	0.62	2.71
S-III, *I*4_1_/acd	*a* = 9.159 (8.594) ^4^ *c* = 4.067 (3.618) ^4^	3.04	2.03
Se-I, *P*3_1_21	*a* = 4.271 (4.366) ^5^ *c* = 4.978 (4.955) ^5^	0	1.69 (1.85) ^6^
Se-II’, *I*4_1_/acd	*a* = 9.737 (9.148) ^7^ *c* = 4.172 (3.626) ^7^	4.25	0.97
Te-I, *P*3_1_21	*a* = 4.371 (4.456) ^8^ *c* = 5.871 (5.921) ^8^	0	0.62 (0.34) ^9^

Experimental data are given in parentheses. ^1^—experimental data from [43], at ambient pressure and temperature; ^2^—experimental value from [44]; ^3^—experimental data from [3], at 3 GPa and 400 °C; ^4^—experimental data from [5], at 12 GPa and 300 K; ^5^—experimental data from [2], at ambient pressure and temperature; ^6^—experimental value from [45]; ^7^—experimental data from [4], at 15.3 GPa; ^8^—experimental data from [1], at ambient pressure and temperature; ^9^—experimental value from [46].

The total electronic energy per periodic cell was calculated with tight convergence threshold 10^−10^ Ha. The tight truncation criteria (10^−10^, 10^−10^, 10^−10^, 10^−10^, and 10^−20^) were used for two-electron integrals of the Hartree–Fock Coulomb and exchange series in the case of sulfur and tellurium. In the case of selenium, the very tight truncation criteria 10^−12^, 10^−12^, 10^−12^, 10^−12^, and 10^−24^ were used. Due to the fact that the technique for obtaining the dependence of energy on the rotation angle *E*(φ) involves the comparison of the energy of structures with very different translation periods, tight truncation criteria should be used to ensure size consistency [35]. Both the translational period and atomic positions have been optimized. The gradient threshold was set equal to 0.00003 Ha∙Bohr^−1^. The Monkhorst–Pack [47] integration method of the one-dimensional Brillouin Zone was used with a 12, 20, and 20 k-point sampling for sulfur, selenium, and tellurium, respectively. These values are sufficient to obtain the results being stable after further increasing of k-mesh.

Phonon harmonic frequencies were obtained via the frozen phonon method [48,49]. In this method, the phonon frequencies are calculated from the forces generated by atomic displacements from equilibrium positions in an appropriate supercell.

The calculation of helically periodic systems was carried out on the basis of the theory of line symmetry groups [33,50,51,52,53] using the algorithm presented in [54]. This algorithm is general and was successfully used by our research team for the calculation of nanohelicenes [54,55,56,57,58], polytwistane [59], helical polyacetylenes [60], nanotubes [58,61,62], and also tellurium nanorods [9].

**Figure 1:**
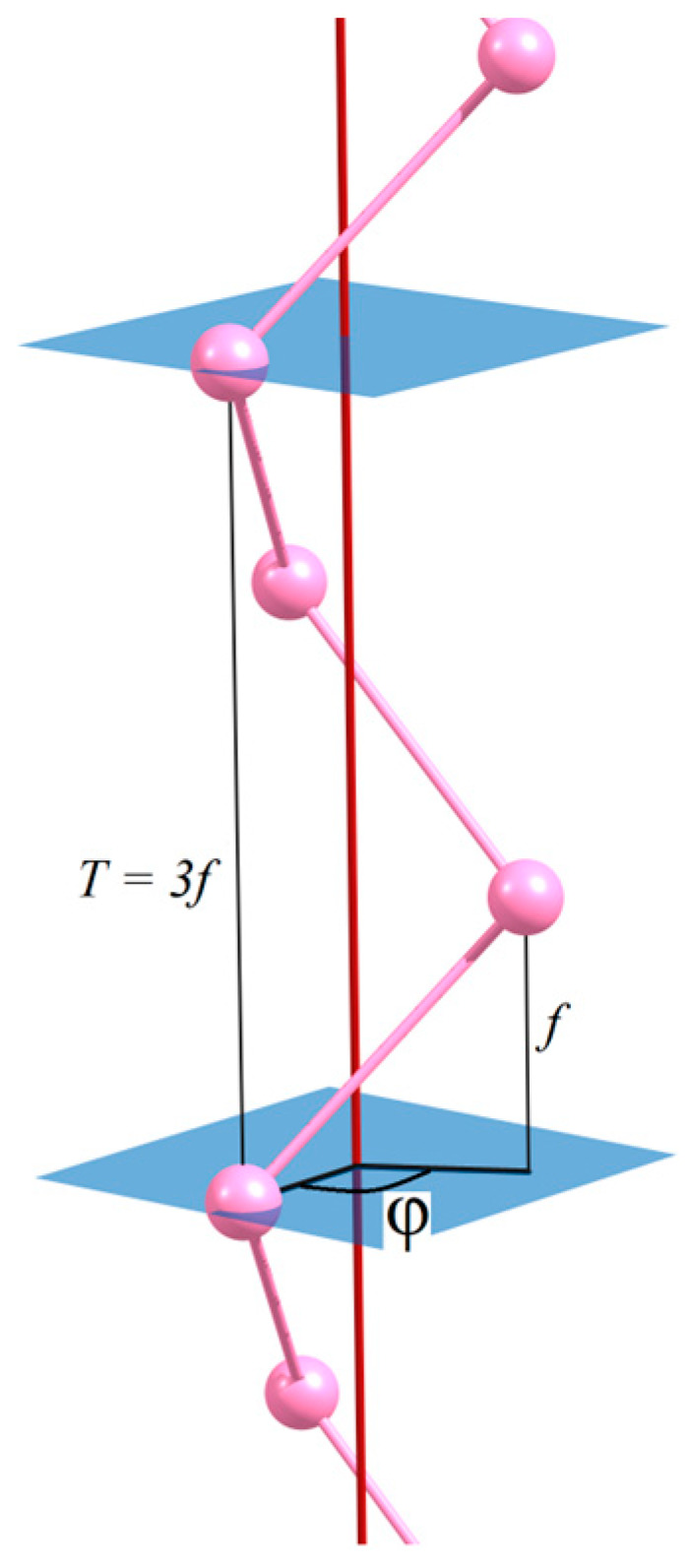
The side view of atomic helical chain with symmetry *L*3_1_21. In this case, *Q* = 3, *q* = 3, *r* = 1, φ = 120°, and *T* = 3*f*.

Among the 13 families of the line symmetry groups [33], there are 2 families (1st and 5th) that allow the order of the helical axis, *Q*, to be any real number, from one to infinity. The symmetry operations generated by the helical axis are generalized translations, *Z* = (*C_Q_*|*f*), that form the abelian group ***Z***. Here, *f* is a partial translation that determines the shift along the *Oz* axis after the monomer has been rotated by the helical rotation angle, φ, defined by Equation (3) (see Figure 1):

(3) $$\varphi =\frac{360{}^{\circ}}{Q}.$$

If *Q* is irrational, then φ is irrational also, i.e., it is impossible to obtain a pure translation by performing a finite number of operations *Z*. Thus, in the case of irrational *Q*, only the helical periodicity remains. If there are no symmetry constraints on *Q*, then *Q* corresponding to the minimum of the potential energy is expected to be an irrational number, since from a mathematical point of view, there are “more” such numbers than rational ones. However, it is known that any irrational number can be represented as the limit of a certain Cauchy sequence of rational numbers. Therefore, although the CRYSTAL17 is a program requiring translational invariance, it can be used to calculate a helically periodic system [54]. Namely, the dependence of the energy per monomer on the rotation angle (torsion energy curve, *E*(φ)) is approximated by interpolation of the data of quantum chemical calculations obtained in rational *Q*, presented in the form

(4) $$Q=\frac{q}{r},\;q>r\;\mathrm{and}\;q,\;r—\mathrm{coprime}.$$

The number of monomers in the periodic cell is determined by *q*. The translational period, *T*, is expressed in terms of partial translation:

(5) T=qf.

Finally, the index *p*, used in crystallographic notation to specify the order of the helical axis (for rational *Q* only), is defined by

(6) rp±1=ql,

where l is some positive integer or zero.

In addition to the symmetry due to the ***Z*** group, the line symmetry group may have an additional symmetry due to the monomer point symmetry group ***P***. In the case of first family, ***P*** = ***C*_n_**. If there are operations generated by a twofold rotation axis, *U*, directed perpendicular to the principal *n* order axis, then ***P*** = ***D*_n_**, and the line symmetry group belongs to fifth family. In the case of atomic helical chains of chalcogens, *n* = 1 and ***P*** = ***D*_1_**. The resulting symmetry of all helical chains will be described by the line symmetry group ***L*** = ***Z***^***D*_1_**, and the whole symmetry group in crystallographic factorization of the calculated structures will be *Lq_p_*22 (even *q*) or *Lq_p_*21 (odd *q*).

Two special cases are also noted. If *Q* = 2 (zigzag chain), symmetry planes appear, and the symmetry group is $p\frac{2_{1}}{m}\frac{2}{m}\frac{2}{a}$ (rod symmetry group No. 22 [63] belongs to the 13th family of line symmetry groups [33]). The linear atomic chain has high symmetry, since *Q* = ∞, the symmetry group of monomer is ***P*** = ***D*_∞h_**, and *f* always coincides with *T* (infinite case of the 11th family of line symmetry group). In this work, the calculation of linear chains was carried out using the subgroup $p\frac{6}{m}\frac{2}{m}\frac{2}{m}$.

For the analysis of the electronic and phonon states in the energy minimum of the helically periodic system, as well as their evolution during torsion deformations, the translational Brillouin zone (TBZ), built on pure translation *T*, is difficult for interpretation. Therefore, the helical Brillouin zone (HBZ), determined by partial translation *f*, is used here. Unlike TBZ, HBZ also exists in the case of irrational values of *Q*. For rational *Q*, both Brillouin zones are possible, i.e., the states can be equivalently classified using the translational wave vector, $k_{t}\in \left(-\frac{\pi }{T},\frac{\pi }{T}\right]$, or helical wave vector, $k_{h}\in \left(-\frac{\pi }{f},\frac{\pi }{f}\right]$. To move from translation classification (CRYSTAL17 output) to helical one, the following formula was used [33,58]:

(7) $$k_{h}=k_{t}+rm\frac{2\pi }{T}+Kq\frac{2\pi }{T},$$

where *m* is a quantum number defined by the isogonal point group ***C****_q_* and the integer *K* is determined from the requirement $k_{h}\in \left(-\frac{\pi }{f},\frac{\pi }{f}\right]$. For convenience, the band pictures presented here show the fractions of the vector $k_{h}$ from 0 (Г point) to 0.5 (X point) along the abscissa axis.

**Figure 2:**
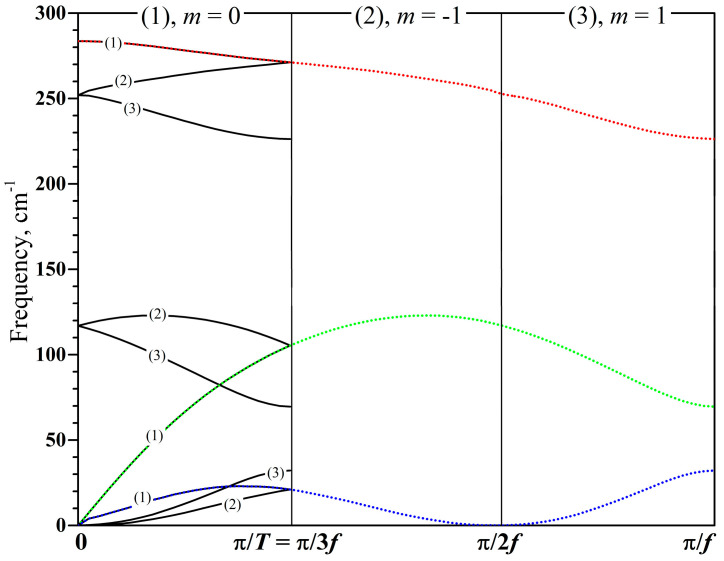
An example of the transformation of phonon dispersion curves from TBZ to HBZ representation. Selenium chain at *L*3_1_21 symmetry (*q* = 3). *m* is a quantum number from Equation (7).

HBZ is in some sense an unfolding of TBZ, so HBZ is *q* times larger than TBZ. Only states with $k_{t}=0$ (Г point) and *m* = 0 will transform to $k_{h}=0$. States with *m* < 0 or *m* > 0 are distributed over the entire HBZ, according to Equation (7). For sufficiently large *q*, it is quite possible to limit ourselves to data from the Г point of TBZ for constructing good approximations of the dispersion curves in HBZ. This is especially important when calculating phonon dispersion curves in HBZ since, in this case, it is sufficient to limit ourselves by calculations only in Г point of TBZ. Nevertheless, in the case of small *q* = 2 ($p\frac{2_{1}}{m}\frac{2}{m}\frac{2}{a}$), 3 (*L*3_1_21), and 4 (*L*4_1_22), the phonon calculations were performed at nonzero TBZ points. To obtain phonon dispersion curves, supercells were constructed by expanding the primitive cell by 20, 40, and 60 times, respectively. Electronic dispersion curves are constructed in a similar way, using information from $k_{t}=0$ at large *q* and additional data from $k_{t}<0$ and $k_{t}>0$ when *q* is small. The comparison of phonon dispersion curves for translational and helical Brillouin zones is given in Figure 2.

For the fifth family of line symmetry groups, there is also a parity with respect to the operations of rotation around the two-fold axes *U*, namely П_U_. This quantum number is relevant only at the Γ and X points of the HBZ, so its presence or absence does not affect the band pictures of the electron or phonon states.

Calculations of axial strains were carried out in a standard way, namely, by optimizing the geometry at a fixed value of relative deformation, τ:

(8) $$\tau =\frac{f}{f_{eq}}.$$

Here, *f* is a given partial translation and *f*_eq_ is its equilibrium value.

These calculations were performed for each chalcogen chain at the minimum of the torsion energy curve. The interval τ∈[0.6, 1.6] and step 0.1 of relative deformations were considered.

## 3. Results and Discussion

### 3.1. Torsion Energy Curves of Chalcogen Chains

Table 1 contains a comparison of the optimized periodic cell parameters of bulk crystals with experimental ones. For the stable phases Se-I and Te-I, the difference between the experimental and theoretical values is no more than 2%. For α-S_8_, this difference is larger but does not exceed 4%. For the metastable high-pressure phases S-II, S-III, and Se-II’, the cell parameters were calculated in the absence of pressure and, therefore, significantly exceed the experimental values.

The calculated values of Δ*E*_form_ for all metastable phases are positive, which corresponds to the experiment and demonstrates the correctness of density functionals and basis sets chosen for the calculation. The stability of the sulfur phases (at zero pressure) decreases in the correct experimental order: α-S_8_ > S-II > S-III. At the same time, the value of Δ*E*_form_ for the S-II phase is very small, which also emphasizes the well-known experimental fact of the existence of different forms of fibrous sulfur at normal pressure [64].

Theoretical estimations of *E*_exf_ of selenium and tellurium atomic chains with non-empirical van der Waals corrections [65] are *E*_exf_(Se, [26]) = 21.8 kJ/mol and *E*_exf_(Te, [26]) = 26.6 kJ/mol. These values are close to our estimations, presented in Table 2 (see Δ*E*_form_ of chains for *L*3_1_21, according to Equation (2)). Values, obtained in [27] using semi-empirical Grimme correction [39], namely *E*_exf_(Se, [27]) = 19.3 kJ/mol and *E*_exf_(Te, [27]) = 26.0 kJ/mol, are also close to our results.

Since the free chain is no longer restricted by the periodicity condition in the direction perpendicular to its symmetry axis, the helical axis order *Q* also becomes a continuous parameter that should be optimized to obtain the minimum of Δ*E*_form_. As can be seen from Table 2, the Δ*E*_form_ at the minimum is always lower than the *E*_exf_ of the selenium and tellurium chains, as well as the Δ*E*_form_ of chains of all chalcogens at any crystallographic value of helical axis order. Tellurium has the highest Δ*E*_form_, and sulfur has the lowest one. The Δ*E*_form_ obtained by us for the given symmetry *L*3_1_21 are arranged in the same order, which agrees with the previously obtained values of Δ*E*_form_(*L*3_1_21) [27]. Thus, the optimization of *Q* preserves the ratio between the formation energies of chalcogen chains obtained for *L*3_1_21.

For all chalcogens, the optimal value of *Q* is in the range from 3 to 4. When moving from sulfur to tellurium, the value of *Q* decreases somewhat. The obtained values correspond to theoretical estimates of the helical rotation angles φ obtained earlier for sulfur [28,29,30], selenium [30,32], and tellurium [9], as well as to the experimental value for selenium [14].

It should be noted that for all chalcogens, Δ*E*_form_(*L*4_1_22) > Δ*E*_form_(*L*3_1_21). This result also confirms the correctness of the density functionals and basis sets chosen for the calculations. It is known from experiments that for all chalcogens, bulk phases consisting of chains with *Q* = 4 have a higher energy than bulk phases consisting of chains with *Q* = 3.

The decrease in energy with increasing order of the helical axis relative to the crystallographic value *Q* = 3 is manifested not only for a single chain, but also for nanorods, i.e., ensembles of chains. This was discovered experimentally [8] and shown theoretically [9] for tellurium nanorods. However, the more chains in a nanorod, the smaller the interval of *Q* in which the chemical structure is preserved. The difference between single chains and nanorods is that single chains preserve chemical bonds in the entire range of rotation angles φ from 0° to 360°, which corresponds to *Q* values from infinity to 1 (see Equation (3)).

In the case of chalcogen atomic chains, the range of φ from 0° to 360° consists of two equivalent intervals, namely (0°, 180°) and (180°, 360°), which correspond to chiral isomers with the same geometric, energetic, and electronic properties. Therefore, we limited ourselves to calculating chalcogen chains only in the first interval [0°, 180°], which also included achiral cases: linear chain with φ = 0° and zigzag chain with φ = 180°.

**Figure 3:**
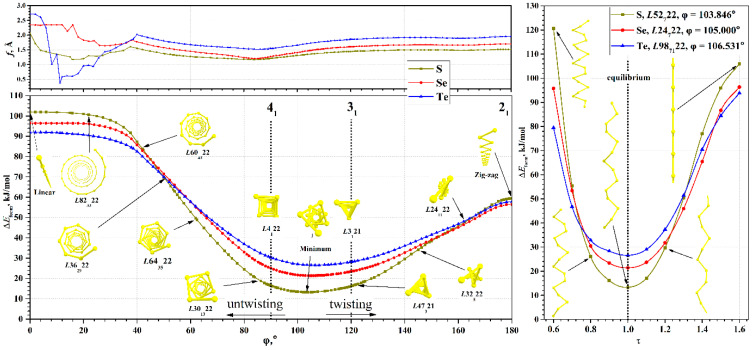
(**Bottom**): Torsion energy curves Δ*E*_form_(φ) of S, Se, and Te chains with top views of atomic structures using sulfur chains as a representative example; (**Top**): Partial translation dependence *f*(φ) of S, Se, and Te chains; (**Right**): Axial energy curves Δ*E*_form_(τ) of S, Se, and Te chains with side views of atomic structures using sulfur chains as representative example.

The torsion energy curves calculated in the range [0°, 180°] are shown in Figure 3. In this range, only one global minimum is observed for all chains, the parameters of which for each chalcogen are given in Table 2. Metastable states are absent, in contrast to the previously studied helical polyacetylenes [60] or nanohelicenes [55,56].

It is convenient to study the evolution of the properties of the chalcogen chains, starting from the minimum energy. Increasing the φ value makes it possible to study the chain properties under torsion stresses corresponding to the *twisting* of the helical chain. Similarly, decreasing the φ will allows us to determine the changes in the properties corresponding to the *untwisting* of the helical chain.

First, we consider the process of twisting of atomic chains. With increasing φ, the energy increases smoothly to values of about 55–60 kJ/mol at 180° (see Table 2). During the twisting, the order of chalcogen chain energies changes and the zigzag chain of sulfur has the maximal energy, while the zigzag chain of selenium has the minimal value.

It should be noted that at φ = 180°, each chalcogen chain under consideration will necessarily have a maximum on the torsion energy curve, since at this point the transition from right to left optical isomers occurs. In other words, this is a transition state. An additional argument is the presence of symmetry planes in the atomic structure at φ = 180°, which makes it distinguished by symmetry. Structures with additional symmetry are extrema—either maxima or minima [66].

In a certain sense, the structure of bulk chalcogen crystals in the form of an ensemble of helical chains is a consequence of the symmetry reduction (discarding planes) and optimization of the order of the helical axes under the additional condition, namely the equality of helical axis order to crystallographic value, *Q* = 3.

Now let us consider the features that arise as φ decreases. In this case, the atomic structure visually untwists, i.e., the radius of the helical chain becomes larger. At the same time, the chain itself in the local sense becomes closer to a linear structure. Such a process at sufficiently small angles φ < 40° leads to unexpected consequences. The parameter *f* ceases to be a good parameter for optimizing geometry, since its change has little effect on the energy. In this region, the main factor possibly becomes the angle between three neighboring chalcogen atoms (bond angle). This is due to the fact that the transition to a linear structure is possible in two ways—either by decreasing φ to zero (the option under consideration), or by increasing the radius of the helical chain to infinity. The uncertainty between these options is clearly visible in the *f*(φ) curve, shown in Figure 3. At values φ > 40°, the curve *f*(φ) is smooth. At φ < 40°, significant noise appears, although the energy curves in this region are smooth.

The relative energy of different chalcogens also changes with untwisting. The linear sulfur chain has the highest energy, and the tellurium chain has the lowest one. The structures at φ = 0° are linear chains and are also structures distinguished by symmetry. For them, there are helical and rotational axes of infinite order, horizontal planes, and an infinite number of vertical planes of symmetry. Accordingly, they are also extrema, namely maxima. Zigzag chains can be considered as structures with partially reduced symmetry (the number of vertical planes decreases to two, and the rotational axis of infinite order disappears) with respect to linear chains. This fact makes it possible to understand why the energy of zigzag chains is lower than the energy of linear ones.

The indicated regularities are common for the considered chalcogens, which is due to the similar structure of the valence shells of their atoms. It is interesting that, although all chains have close dependences of the torsion energy, the bulk crystals of stable phases have a qualitatively different structure. Bulk crystals as a set of chains are most favorable for selenium and tellurium, while for sulfur such a crystal is metastable.

The dependences of the Δ*E*_form_(τ) of chalcogen chains at energy minima are shown in Figure 3. In the region of small strains, the Δ*E*_form_(τ) dependence is close to quadratic. Deviations from quadraticity are expectedly large in the case of maximal and minimal τ. Compressions for which τ < 0.6 lead to the formation of bonds between the coils of the chains and the structure ceases to be a single helical chain. Therefore, such compressions were not considered here. Extreme extensions, τ > 1.6, lead to a structure visually indistinguishable from linear chains. However, even in a strongly extended chain, the distance from the chalcogen atom to the main axis is not zero, since the symmetry group is only a subgroup of the true symmetry group of the linear chain. Since the linear chain is at the energy maximum, any decrease in symmetry will lead to a decrease in energy at a small displacement of the atoms, regardless of the value of the partial translation.

### 3.2. Electronic Structure of Chalcogen Chains: Torsion and Axial Deformations

**Figure 4:**
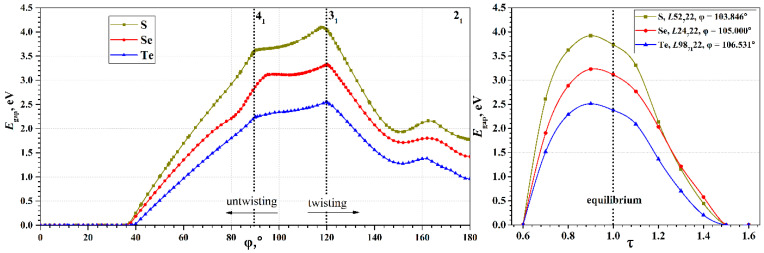
(**Left**): The *E*_gap_(φ) curves of S, Se, and Te chains; (**Right**): The *E*_gap_(τ) curves of S, Se, and Te chains.

An important characteristic of the chains, determining their use in practical applications, is the electronic band gap, *E*_gap_, which defines electron transport properties of nanostructures. The wide range of possible torsion deformations of chalcogen chains leads to a wide range of possible band gap width, and, consequently, to greater possibilities for tuning the electronic properties (see Figure 4). The *E*_gap_(φ) curves have maxima in the region of φ~118–120°, and zero values in the region of φ < 37°. All three chalcogens exhibit qualitatively very similar dependences; the difference is only in the maximal value of the *E*_gap_. It is expected that sulfur has the largest maximum, and tellurium has the smallest, which corresponds to the order of *E*_gap_ observed for bulk crystals (Table 1).

It is interesting that the minima on the *E*_form_(φ) curves do not appear on the *E*_gap_(φ) curves, i.e., they do not correspond to any special φ point. In addition, unlike the *E*_form_(φ), the *E*_gap_(φ) curves have a much more complex dependence on the φ. In the 150° region, they demonstrate a local minimum, which is obviously also present in the region near 180°. In the 90–95° and 118–120° regions, there are cusp points. An explanation of such behavior of the *E*_gap_(φ) was given in the case of a tellurium chain considered in the φ∈[86.400°,127.059°] interval [9]. According to [9], such behavior is due to the evolution of individual helical electronic bands. We consider them in more detail.

**Figure 5:**
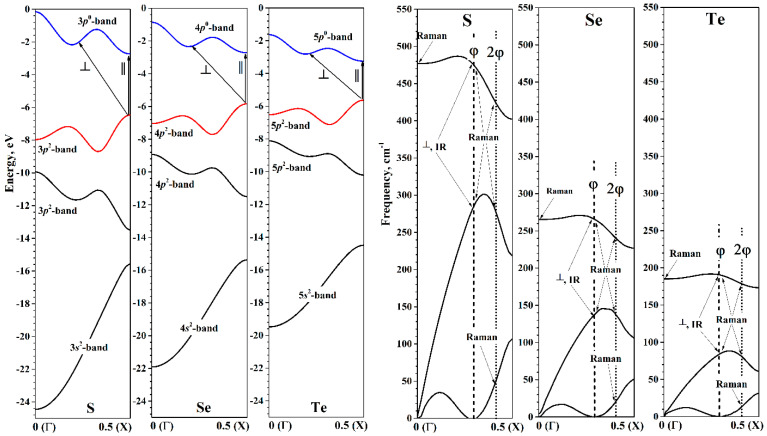
Helical bands of S, Se, and Te chains in HBZ at energy minima. (**Left**): Electronic states. Upper valence and lower conduction helical bands are red and blue, respectively. Allowed optical transitions for parallel (∥) and perpendicular (⊥) polarizations are shown by arrows; (**Right**): Phonon states. Allowed IR and Raman transitions are shown by arrows.

The chalcogen chain’s electronic structure in HBZ energy band description near the energy minimum is shown in Figure 5. It is worth noting the convenience of the HBZ picture. Since the monomer at any *Q* value consists of only one chalcogen atom, each of the valence orbitals of the atom will produce exactly one corresponding helical band at any *Q*. As is known, the valence shell of chalcogen atoms is given by the general formula *ns*^2^*np*^4^. Two of the three valence *p*-orbitals are filled. This means that when the chain is formed, both the upper valence and lower conduction helical bands will be formed mainly by valence *p*-orbitals.

In the region of energy minima, all the studied chalcogen chains are semiconductors, which have *E*_gap_ greater than the corresponding values of bulk crystals. The top of the valence helical band and the bottom of the helical conduction band are located at the edge of the HBZ (X point), so that in the region of energy minima, the chalcogen chains are direct-gap semiconductors. The decrease in the band gap width when going from sulfur to tellurium occurs mainly due to the increase in the top of the helical valence band. The decrease in the bottom of the helical conduction band occurs only when going from selenium to tellurium.

**Figure 6:**
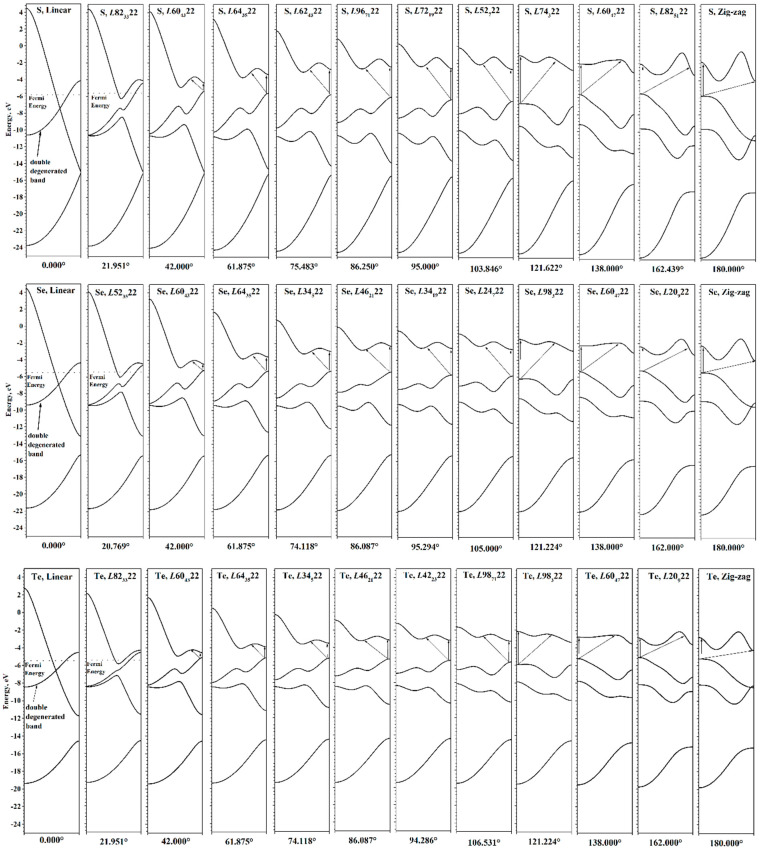
Evolution of electronic helical bands of S, Se, and Te under torsion. Allowed optical transitions are shown by arrows.

The change in the helical bands during untwisting (i.e., deformation from energy minimum to 0°) is shown in Figure 6. For practical applications, it is most important to analyze the changes in the upper valence and lower conduction helical bands. During untwisting, the valence band energy near Г point (Г-end) decreases, and near the X point (X-end) increases. The Г-end of the conduction helical band successively ascends during untwisting. The X-end of the conduction band ascends during untwisting to approximately φ~80°, and then begins to descend, gradually approaching the X-end of the valence helical band. It is also worth noting the special behavior of the middle part (“dip”) of the conduction helical band, which descends much faster during untwisting. This leads to the fact that after φ~90–93°, this dip becomes the bottom of the conduction helical band, and accordingly, *E*_gap_ is no longer defined as a direct transition.

As it descends further, this dip at φ < 38–40° drops below the top of the valence helical band, resulting in a zero band gap and semi-metallic conductivity, since the helical bands do not intersect until reaching φ = 0° (linear chain). In the electronic structure of linear chains, the parts of the helical bands are sticking together, forming one doubly degenerate π-band, composed of *p_x_*- and *p_y_*-orbitals. The dispersion of this band is similar to that of the helical band generated by the *ns*-orbitals. The remaining helical band is the σ-band, composed of *p_z_*-orbitals. Since the helical bands transform over different irreducible representations of the point symmetry group of the linear chain, ***D*_∞h_**, their intersection becomes possible. The unique electronic structure of linear chains is a direct consequence of the presence of additional elements of symmetry, namely, the vertical reflection planes. The presence of these planes leads to the appearance of an additional quantum number, the parity with respect to the reflection in the vertical plane, Π_v_ [33]. Unlike Π_U_, the parity Π_v_ exists also for helical wave vectors inside HBZ, which leads to the possibility of band degeneracy observed in the electronic structure of linear chains.

Considering the changes in helical bands during untwisting as a whole, it is easy to notice that the transition from a linear chain to the optimal helical structure is accompanied by a “pushing out” of the helical bands caused by a torsion-induced spontaneous symmetry breaking. The greater the distortion of the symmetry of the helical chain relative to the symmetry of the linear chain, the greater the pushing out of the helical bands.

Now consider the evolution of the helical bands in the HBZ during twisting the chains from the minimum energy to φ = 180° (Figure 6). The X-end of the upper valence helical band successively decreases, and the part of the valence helical band near the Г-end rises. This leads to the result that in the region of φ~118–120°, the maximum of the valence helical band jumps to the region of Г-point of HBZ and remains there up to 180°. This is manifested by the appearance of a cusp in the *E*_gap_ curve, explained by us earlier when considering the tellurium chain [9]. Now it is clear that the appearance of a cusp in this region is common to all chalcogens and has the same cause.

The conduction helical band has a more complex behavior during twisting. The main changes occur in the inner region of the band, only slightly changing the Г and X ends of the helical band. The bottom of the conduction helical band remains at point X of HBZ. Thus, after φ~118–120° and up to the zigzag chain, the top of the valence helical band and the bottom of the conduction helical band are at different points of the HBZ and the electronic band gap is no longer defined as a direct one.

In zigzag chains, due to symmetry, the upper valence helical band consists exclusively of *p_y_* orbitals directed perpendicular to the σ_v_ plane and forming π bonds between the chalcogen atoms in the zigzag chains. This band consists of “ungerade” states relative to the σ_v_, while the other two *p* bands (the lower valence and conduction helical bands) consist of “gerade” states. For the upper and lower valence *p* bands, a crossing possibility appears, which occurs at some point near the HBZ edge. Note that in our calculations, zigzag chains remain semiconductor structures with a nonzero value of the *E*_gap_.

**Figure 7:**
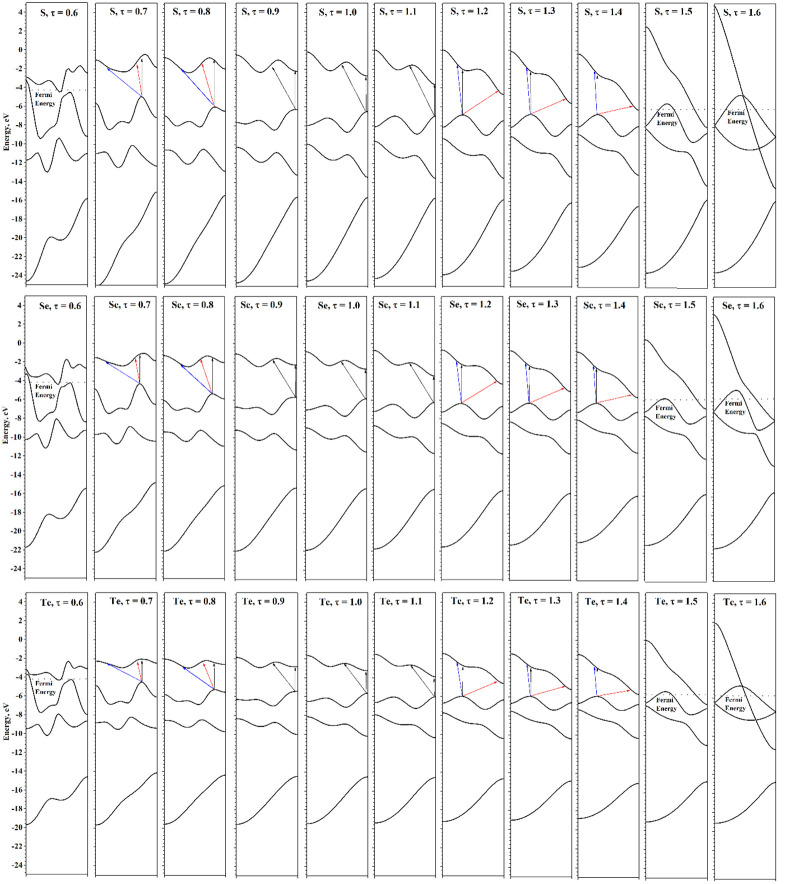
Evolution of electronic helical bands of S, Se, and Te under axial strain. Allowed optical transitions are shown by arrows.

Next, consider the evolution of the electronic structure under the axial strains. Changes in the atomic structure of chains under axial strain are clearly manifested in the evolution of electronic helical bands (see Figure 7). Under compression, the dispersion of helical bands becomes more pronounced and sharper, which is due to the ever-increasing interactions between the coils. Under tension, the situation is reversed: the bands become smoother, gradually passing to a band structure that has a certain similarity with the band structures described earlier for linear chains. However, although under sufficient tension the structures are visually indistinguishable from linear chains, their symmetry groups are the same as for the equilibrium structures. So, the previously mentioned small differences in the distance from the chalcogen atom to the main axis lead to qualitative differences between the electronic structure of chains stretched at minimum energy and linear chains at maximal symmetry.

In the case of chains obtained by extreme stretching from the energy minimum, the σ band composed of *p_z_*-orbitals has a form similar to the σ band of linear chains. However, the *p_x_* and *p_y_* orbitals do not form a doubly degenerate π band, but form two helical bands that join at the Γ and X points of the HBZ. The bands have a simple concave and convex shape; the degree of divergence between them is determined by the interaction between the chalcogen atoms. At stretching τ = 1.6, the interaction is strong enough, causing a significant dispersion of these helical bands. With further stretching, the interactions between the atoms will decrease, and the dispersion of the *p* bands will decrease also. In the limit of infinite stretching, the chains will consist of infinitely separated atoms, and their helical band structure will obviously consist of two flat helical bands—a non-degenerated *s* band and a triply degenerated *p* band.

### 3.3. Phonon Dispersion Structure of Chalcogen Chains: Torsion Deformaions

The phonon dispersion at the minimum of energy in the helical factorization is shown in Figure 5. Since the monomer of all the considered chains consists of one atom, there will always be three phonon dispersion curves in HBZ. Similarly to the electron helical bands, the phonon helical bands do not intersect, since at a given *k_h_* they are transformed according to the same irreducible representation. The first two helical bands correspond to acoustic modes, and the third band is an optical one. For the three phonon curves of all the considered chalcogen chains, there are no imaginary frequencies. This confirms the stability of the chalcogen chains in the minima of the torsion energy curves.

Consider the first acoustic helical band in more detail. This band “touches” the abscissa axis at two points (Figure 5). The first point is the Г point of the HBZ. According to Equation (7), this point corresponds to the irreducible representations *A*_1_ or *A*_2_ at Г point of the TBZ. At the same time, the acoustic band touches the abscissa axis in the region $k_{h}\sim \frac{1}{3}$. More precisely, this point always refers to the irreducible representation *E*_1_ at Г point of TBZ, which is responsible for the translations of the chain as a whole in directions perpendicular to the main axis. The exact value of *k_h_*, to which *E*_1_ passes, depends on the value of φ:

(9) $$k_{h}(E_{1})=\pm \varphi \frac{0.5}{180{}^{\circ}}.$$

Here, it is assumed that φ is in the range from 0° to 180°. In the HBZ pictures, the abscissa axis is shown in the range from 0 to 0.5, so only the positive value of the pair defined by Equation (9) is shown in these pictures.

Thus, the first acoustic helical band has a characteristic two-part view. The first part is a convexity in the region from the Г point to $k_{h}(E_{1})$. The second part smoothly increases from zero value at $k_{h}(E_{1})$ to some value at the X point of HBZ.

**Figure 8:**
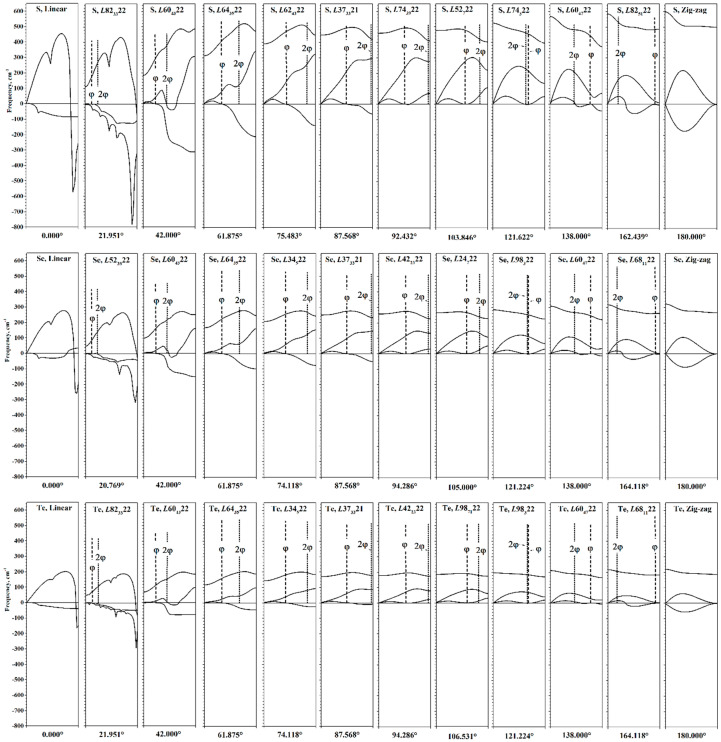
Evolution of phonon helical bands of S, Se, and Te under torsion. Allowed IR and Raman transitions for perpendicular polarization of light are shown as φ and 2φ.

**Figure 9:**
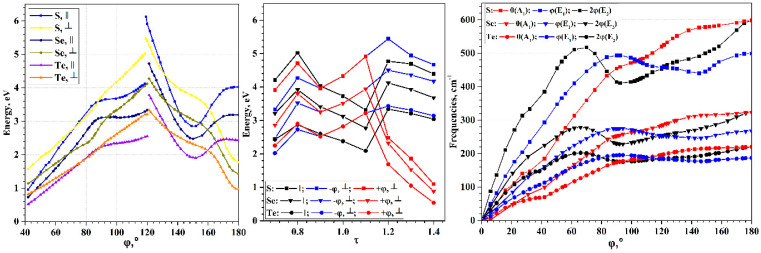
Evolution of allowed transitions of S, Se, and Te. For designations of transitions see Equations (9)–(13). (**Left**): Electronic transitions under torsion deformations; (**Middle**): Electronic transitions under axial deformations; (**Right**): Vibrational transitions of optic phonon helical bands under torsion deformations.

For further discussion, we also define the value of *k_h_*, to which the irreducible representation *E*_2_ at Г point TBZ transforms:

(10) $$k_{h}\left(E_{2}\right)=\pm 2\varphi \frac{0.5}{180{}^{\circ}}+K,$$

where *K* is some integer required if the value of $k_{h}\left(E_{2}\right)$ is outside the domain of definition. For convenience, in the HBZ pictures (Figure 5, Figure 8 and Figure 9 the $k_{h}\left(E_{1}\right)$ is denoted as φ, and $k_{h}\left(E_{2}\right)$ as 2φ.

The phonon dispersion curves of the chalcogen chains in the region of the energy minima are qualitatively similar. The differences are expectedly due to the difference in the atomic masses of chalcogens. The phonon dispersion curves of the selenium chains are very close to the results obtained on the basis of calculations by the MP2 method in the cluster model [32]. It can be assumed that the shape of the phonon dispersion curves in the helical factorization has a qualitatively similar form in the case of any monatomic helical chains. In particular, the shapes of the phonon dispersion curves for oxygen helical chains [67] are also close to our results for S, Se, and Te helical chains.

Although the phonon spectrum has physical sense only at the equilibrium point, its calculation at different values of φ can nevertheless provide information about the behavior of the chalcogen chains during torsion (see Figure 8).

The evolution of the phonon helical bands during torsion is most pronounced in the first acoustic phonon helical band. First, consider its evolution during untwisting. At the first stage, the X end of this helical band descends, reaching zero values at φ~88–91°. With further torsion, the second part of the acoustic band (from $k_{h}\left(E_{1}\right)$ to the X point of HBZ) is now entirely in the region of imaginary frequencies, showing that the chains are no longer stable. However, the first part of the acoustic helical band (from the Г point to $k_{h}\left(E_{1}\right)$) has positive frequencies.

For φ < 40°, qualitative changes occur in the phonon spectrum. For the second acoustic band, an interval of *k_h_* appears, which has imaginary frequency values. Note that only for interval φ < 40°, the problem of the *f* parameter optimization arises, as shown in Figure 3. With further untwisting, the second acoustic band quickly goes into the region of imaginary frequency values, and imaginary values of the optical helical band appear near the X point of HBZ. In addition, all helical bands cease to be smooth—sharp peaks appear. The number of these peaks is different for different helical bands.

In the phonon dispersion picture for linear chains, which are the final result of untwisting, the acoustic bands merge into a double degenerate band, which has negative values at *k_h_* > 0 (in the case of a selenium, there is a small region of real positive values located closer to the X-end of the HBZ). The appearance of two phonon helical bands with imaginary frequency values indicates that displacement in any direction perpendicular to the main axis will result in a decrease in energy. At Г point, all three phonon bands are connected, and frequency has a zero value, since they relate to translational degrees of freedom.

Next, we consider the evolution of the phonon dispersion curves during twisting. Up to φ~130°, the phonon bands retain their shape, and there are no imaginary frequencies. After 130°, regions with imaginary frequency values begin to appear in the first acoustic helical band. It should be noted that the changes in this band during twisting have a qualitatively different character in relation to the changes during untwisting. In particular, imaginary values appear not only in the region from $k_{h}\left(E_{1}\right)$ to the X point of HBZ, but also in the region from the Г point to $k_{h}\left(E_{1}\right)$. The shape of this band becomes more complicated.

During twisting, the $k_{h}\left(E_{1}\right)$ point shifts to the X point of HBZ. For zigzag chains, $k_{h}\left(E_{1}\right)$ exactly coincides with the X point of HBZ, and the first acoustic band consists of imaginary frequencies. This phonon band corresponds to shifts in atoms from the σ_v_ plane of the zigzag chain. Both acoustic bands are almost the same shape and are approximately symmetrical relative to the abscissa axis. Both of these bands are connected at Г and X points of HBZ.

### 3.4. Electronic and Vibrational Transitions: Torsion and Axial Deformations

According to the selection rules, three types of transitions are allowed in electronic optical absorption spectra depending on the direction of light polarization [33,68]. In the case of parallel polarization, designated in Figure 5, Figure 6 and Figure 7 and Figure 9 by “∥”, only vertical transitions in HBZ are allowed:

(11) $$∆k_{h}(∥,0)=0.$$

In the case of perpendicular polarization, denoted by “⊥”, two types of transitions are allowed:

(12) $$∆k_{h}(⊥,+\varphi )=\varphi \frac{0.5}{180{}^{\circ}}+K,$$

(13) $$∆k_{h}\left(⊥,-\varphi \right)=-\varphi \frac{0.5}{180{}^{\circ}}+K.$$

Here, *K* is some integer required if the $∆k_{h}$ results in *k_h_* outside the domain of definition. The transitions $∆k_{h}(∥,0)$, $∆k_{h}(⊥,+\varphi )$, and $∆k_{h}\left(⊥,-\varphi \right)$ correspond to direct transitions in TBZ, i.e., $∆k_{t}=0$.

The transitions defined by Equations (12) and (13) in general have different energies and oscillator strengths, which gives different peaks in the electronic absorption spectra. However, if the transition occurs from the Γ or X points of the HBZ, then the points to which the transitions (⊥,+φ) и (⊥,−φ) occur will be equivalent reciprocal lattice points and will give the same energies and oscillator strengths. In this case, the absorption of perpendicularly polarized light can be described only by Equation (12).

The allowed electronic transitions near the energy minima of chalcogen chains are shown in Figure 5, and their numerical values are presented in Table 3. Since the top of the valence band is at the X point of HBZ, only one transition will manifest itself for perpendicular polarization. Since the bottom of the conduction band is also at the X point, the direct transition will coincide with the *E*_gap_ from Table 2. Therefore, at the energy minimum, the transition (⊥,φ) has higher energy than the transition (∥,0) for all chalcogen chains. Also note that the energies of both the direct and indirect allowed transitions are expectedly reduced in the series S, Se, and Te.

Changes in the energies of allowed electronic transitions under torsion deformations are shown in Figure 9. In the region φ~119–120°, breakpoints appear on the optical transition curves. In this region, the top of the valence helical band moves from the X to the Г point of HBZ. Therefore, passing through the breakpoint leads to sharp changes in energy for both direct (∥,0) and indirect $\left(⊥,\varphi \right)$ transitions.

The energy minima of chalcogen chains are in the region of φ < 119–120°, so chains untwisting will only lead to smooth changes in the transition energies. Note the general tendency to decrease the energy of both types of transitions, which correlates with the *E*_gap_(φ) curve (Figure 4). However, the transition energy curves have a qualitatively different shape, so in the region of φ~70–90° the (∥,0) and $\left(⊥,\varphi \right)$ transitions have the same energy. Moreover, for selenium chains, the $\left(⊥,\varphi \right)$ energy is lower than the (∥,0) energy. With further untwisting, the (∥,0) energies again become lower than the $\left(⊥,\varphi \right)$ energies. At φ < 40°, optical transitions were not considered, since in this region, chalcogen chains exhibit metallic properties.

When the chains are twisted from the energy minimum, the already noted qualitative change in the transition energy curves occurs. It is caused by a change in the location of the top of valence helical band (see transitions in Figure 6). The vertical transition (∥,0) becomes higher in energy than the transition $\left(⊥,\varphi \right)$. When twisted further, the (∥,0) energy quickly decreases, but starting from φ~150°, it begins to increase. The $\left(⊥,\varphi \right)$ energy decreases when twisted. The region of φ from 130° to 160° is a region where the (∥,0) energy is lower than the $\left(⊥,\varphi \right)$ energy.

Torsion deformations lead to significant changes in the optical transition energies. However, both the Г and X points of HBZ are special points. Therefore, in the entire possible range of torsion deformations of chalcogen chains, the difference between the transitions (⊥,+φ) and (⊥,−φ) is not observed. However, the situation changes qualitatively under axial deformations. According to Figure 7, under deformations τ < 0.9 or τ > 1.1, the top of valence helical band shifts from the X point of HBZ to a point located inside HBZ. According to Equations (12) and (13), indirect transitions in this case will occur to nonequivalent HBZ points, therefore, the transitions (⊥,+φ) and (⊥,−φ) have different energies and oscillator strengths (see the transition directions in Figure 7 and the transition energies in Figure 9). Stretching leads to a significant decrease in the (⊥,+φ) energy, but compression leads to an increase in this value. The different relationship between the (⊥,+φ) and (⊥,−φ) energies, observed during compression and stretching, is due to the different positions of the top of valence helical band. During compression, the top of the valence helical band is near the X point of the HBZ. During stretching, the top of valence helical band moves to the region near the Г point of the HBZ. The latter also leads to a sharp change in the energy of the direct transition (∥,0).

It can be assumed that axial deformations at any possible value of the φ lead to a divergence of the (⊥,+φ) and (⊥,−φ) transitions, which are the same at the equilibrium *f* value. In other words, the torsion deformation of the chalcogen chains changes the energy of transition (⊥,φ), while the axial deformation separates this transition into positive and negative components (⊥,+φ) and (⊥,−φ). This fact may prove to be extremely promising for use in the design of opto-mechanical nanodevices.

Now we estimate the frequencies of allowed vibrational transitions of chalcogen chains. The selection rules for infra-red (IR) spectra are determined by the direction of polarization of the incident light [33,70]. With parallel polarization, vertical transitions corresponding to $k_{h}=0$ are allowed. It was noted earlier that these transitions correspond to the excitation of vibrations that transform according to the irreducible representations *A*_1_ or *A*_2_ at the Γ point of the TBZ. Of these two representations, only *A*_1_ is allowed for IR spectra. In the case of perpendicular polarization, only vibrations that transform according to the irreducible representation *E*_1_ from the Γ point of the TBZ can be active in the IR spectra (see Equation (9)).

The selection rules for Raman spectra are determined by both the direction of incident and scattered light [33,70]. In the case of parallel polarization of incident light, vertical transitions $k_{h}=0$ and the irreducible representation *A*_2_ at the Г point of TBZ are allowed. In the case of perpendicular polarization, transitions at the points $k_{h}(E_{1})$ and $k_{h}(E_{2})$ are allowed, determined according to Equations (9) and (10), respectively.

Table 3 lists the values of frequencies possible according to the selection rules. Not all of them are realized in IR or Raman spectra. For example, the vibrations ($∥,\;0\left(A_{2}\right)$) allowed for IR spectra have zero or close to zero values, corresponding to translational degrees of freedom. Therefore, in the IR spectra of chalcogen chains, only two transitions at the $k_{h}(E_{1})$ points will be visible, which was noted earlier for selenium chains [32].

Raman spectra can potentially exhibit a much larger number of peaks. In particular, the ($∥,\;0\left(A_{1}\right)$) transition is possible. This transition is manifested in the experimental Raman spectra of selenium chains [69]. The experimental value of the ($∥,\;0\left(A_{1}\right)$) transition frequency is close to the value obtained by us and to the result of another theoretical study of selenium chains [32] (see Table 3). For Raman spectra corresponding to perpendicular polarization, two transitions of the ($⊥,\varphi (E_{1})$) type and three transitions of the ($⊥,\;2\varphi (E_{2})$) type are possible. In the case of selenium chains, our results are very close to the theoretical results of [32].

Since the first and second acoustic phonon helical bands can descend to the region of negative frequency values during torsion deformations, only the change in the allowed transitions belonging to the optical phonon helical bands is shown in Figure 9. The directions of the transitions are shown in the phonon dispersion pictures in Figure 8. In the region of energy minima, the values of the frequencies of the optical phonon helical band of the ($∥,\;0\left(A_{1}\right)$) and ($⊥,\;\varphi (E_{1})$) types are very close and higher than the values of the ($⊥,\;2\varphi (E_{2})$) type frequencies. However, under the torsion deformation, this order repeatedly changes. In fact, on the dependence of the frequency values, there are regions corresponding to each possible ordering by energy among the allowed transitions.

Let us note two extreme cases of torsion deformations that determine the behavior of the frequencies of allowed vibrational transitions. For a linear chain, the allowed transitions of all types obviously coincide and, according to the phonon dispersion pictures in Figure 8, are equal to zero. This leads to a general tendency to decrease in the frequencies of allowed transitions during untwisting, which is observed in Figure 9. For a zigzag chain, the transition of the type ($⊥,\;2\varphi (E_{2})$) becomes equivalent to the direct transition ($∥,\;0\left(A_{1}\right)$). Thus, when twisting the chains, the frequency curves of these types connect at φ = 180°. Transitions of the type ($⊥,\;\varphi (E_{1})$) are characterized by a lower frequency value.

## 4. Conclusions

According to the results of our first principles calculations, the torsion energy curves of considered free chalcogen chains (S, Se, and Te) have a single minimum in the region of helical rotation angle φ~103–107°. The phonon dispersion curves in this region do not have imaginary frequencies, which confirms the stability of chalcogen chains. Linear and zigzag chains correspond to extreme points on the torsion energy curves, since they additionally have symmetry planes. It can be assumed that the optimal structure of chalcogen chains as helical ones is obtained from linear or zigzag chains due to a sequential symmetry breaking.

Unlike the torsion energy curves, the dependence of the electronic band gap on the helical rotation angle, *E*_gap_(φ) has a complex form, including both semiconductor and semi-metallic states. In the region of φ~119–120°, a cusp is observed in the *E*_gap_(φ) curve. The appearance of this cusp is a result of changing the top of the valence helical band from the X to Г point of HBZ. The reason for the cusp, as well as other features of the electronic structure of chalcogen chains, becomes obvious when analyzing the evolution of the topology of electronic helical bands under torsion or axial deformations.

The energies of the optical electronic transitions for parallel (∥,0) and perpendicular (⊥,φ) polarization of light also change over a wide range under torsion deformation. The mentioned change in the position of the top of valence helical band leads to a breakpoint in the optical transition energy curves.

While torsion deformations of chalcogen chains change only the energy value of indirect (⊥,φ) transitions, axial deformations separate these transitions into positive and negative components, namely (⊥,+φ) and (⊥,−φ) transitions. This fact creates the possibility of combining torsion and axial deformations. This combination is extremely attractive for the design of opto-mechanical nanodevices.

Analysis of phonon dispersion curves during torsion deformations allows us to trace the regular manifestation of the atomic structure instability. Untwisting into a linear chain leads to negative values of two acoustic helical bands, since any combination of atomic displacements in the horizontal plane leads to a decrease in energy. Twisting into a zigzag chain shifts only one mode to the region of imaginary frequency values. Thus, only atomic displacements that move out of the vertical plane will lead to a decrease in energy.

## Figures and Tables

**Table 2:** Calculated geometrical, energetic, and electronic properties of S, Se, and Te atomic chains.

Chain	*Q*	φ, °	*f*, Å	*r*^1^, Å	*d*^2^, Å	Δ*E*_form_, kJ/mol	*E*_gap_, eV
S, linear	∞	0	2.080	0	2.080	101.9	0
S, *L*4_1_22	4	90	1.208	1.176	2.056	16.4	3.62
S, minimum	3.465	103.889	1.347	0.990	2.061	13.3	3.74
S, *L*3_1_21	3	120	1.446	0.852	2.066	16.3	4.04
$S,\;L\frac{2_{1}}{m}\frac{2}{m}\frac{2}{a}$	2	180	1.518	0.733	2.111	59.4	1.78
Se, linear	∞	0	2.356	0	2.356	96.5	0
Se, *L*4_1_22	4	90	1.270	1.387	2.337	25.0	2.87
Se, minimum	3.423	105.185	1.484	1.140	2.341	21.4	3.11
Se, *L*3_1_21	3	120	1.608	0.987	2.346	23.5	3.33
$\mathrm{Se},\;L\frac{2_{1}}{m}\frac{2}{m}\frac{2}{a}$	2	180	1.704	0.840	2.393	56.5	1.42
Te, linear	∞	0	2.726	0	2.726	92.0	0
Te, *L*4_1_22	4	90	1.545	1.573	2.708	30.3	2.24
Te, minimum	3.377	106.608	1.746	1.295	2.713	26.6	2.38
Te, *L*3_1_21	3	120	1.855	1.147	2.718	28.2	2.55
$\mathrm{Te},\;L\frac{2_{1}}{m}\frac{2}{m}\frac{2}{a}$	2	180	1.957	0.974	2.762	57.8	0.96

^1^—radius, i.e., distance from atom to *Oz* axis; ^2^—bond length.

**Table 3:** Electronic and vibrational allowed transitions of S, Se, and Te chains at energy minima.

	Electronic, eV		Vibrational (IR, Raman), cm^−1^			
	(∥,0)	(⊥,φ)	$∥,0(A_{1})$	$∥,0(A_{2})$	$⊥,\varphi (E_{1})$	$⊥,2\varphi (E_{2})$
S	3.74	4.33	477	0; 3	0; 281; 477	58; 270; 420
Se	3.11	3.51	265 (262) ^1^ (259) ^2^	0; 4	0; 137 (136) ^1^; 266 (270) ^1^	25 (33) ^1^; 133 (136) ^1^; 237 (247) ^1^
Te	2.38	2.82	184	0; 3	0; 84; 191	15; 81; 178

^1^—computational data from [32]; ^2^—experimental data from [69].

## Data Availability

Data are contained within the article.
